# Nonsynostotic Plagiocephaly: Prevention Strategies in Child Health Care

**DOI:** 10.3390/jcm9123946

**Published:** 2020-12-05

**Authors:** Freda Lennartsson

**Affiliations:** Department of Pediatrics, The Sahlgrenska Academy, Institution for Clinical Sciences, University of Gothenburg, 416 85 Gothenburg, Sweden; freda.lennartsson@gmail.com

**Keywords:** assessments, child health, education, infants, intervention, nonsynostotic plagiocephaly, nurses’ instruction, parents, prevention, reversal

## Abstract

The dissertation, comprising a clinical intervention and three supporting studies, aimed to assess if it is possible to prevent nonsynostotic plagiocephaly while promoting safe infant sleeping practices. Five individuals were trained to assess cranial asymmetry and then reliability-tested; the interpreted results indicate substantial strength of rater-agreement. Intervention participants were allocated to group. Only intervention group nurses participated in the continuing education on plagiocephaly developed for nurses. A survey compared information intervention and control group parents received from nurses; intervention group parents were significantly more aware of recommendations than the controls. Nurse education was evaluated by asking intervention and control group nurses and parents two open-ended questions; the intervention group reported new re-positioning strategies. The effect of the intervention on cranial shape was evaluated by assessing asymmetry at 2, 4, and 12 months (176 intervention group; 92 controls). It was nine times more common that cranial asymmetry at two months reversed by four months when parents were aware of written recommendations from their nurse (OR = 9.09 [0.02; 0.48], *p* = 0.004) when adjusted for group. An infant’s risk of asymmetry persisting until 12 months was significantly reduced in the intervention group (RR = 0.35 [0.13; 0.94], *p* = 0.03). Preventing brachycephaly was difficult. Conclusions: the assessors were considered reliable; educating nurses promoted the integration of new recommendations in practice; the intervention was associated with early reversal of nonsynostotic plagiocephaly.

## 1. Introduction

Nonsynostotic plagiocephaly (NSP) is acquired cranial asymmetry that develops from pressure which occurs when an external force is regularly applied to an area of an infant’s cranium over a period of time [1]. The contact force generated between the cranium and resting surface resists growth in the area where there is contact and displaces growth to areas with no resistance. This process is similar to how a pumpkin flattens as it grows—it cannot expand into the ground and therefore grows along it [2]. There are three main groups of NSP: plagiocephaly-skewed occipital flattening, brachycephaly-symmetric occipital flattening, and combined plagiocephaly/brachycephaly [3].

Prevalence is difficult to determine. In the first weeks postpartum, it is difficult to differentiate between pre-natal NSP and cranial molding from the birth process; no study has established when cranial molding stops and post-natal NSP begins [4]. In addition, prevalence is a measure which is calculated at one point in time, but NSP is not static. It can develop, reverse, and develop again [5]. The prevalence seems to rise during the first four months and then gradually decreases [6], so age differences are important to consider when calculating prevalence. According to a systematic review, the point of prevalence may be as high as 22.1% at seven weeks and as low as 3.3% at two years [7].

Coinciding with the “back to sleep” campaign in the early 1990s, when parents were recommended to place their infants supine for sleep to prevent Sudden Infant Death Syndrome (SIDS), there was a sharp increase in NSP referrals to craniofacial centers [8,9,10]. A noticeable asymmetric face is often considered less attractive, which can lead to psycho-social developmental consequences [11]. Severe NSP in the childhood years can lead to teasing, poor self-conception, and teacher bias [12]. Possible sequelae of NSP beyond the psycho-social concerns are being researched. A study evaluating neurologic profiles of infants 4 to 13 months old found significantly more altered tone—deflecting abnormally high and low tone—in infants with NSP compared to infants without [13]. A study comparing three-year-old children who had been operated on and not operated on for their NSP, found that 25% of the 12 children who had not been operated had severe receptive language skill problems. [14]. A study comparing the development of 36-month-old children found that the 224 children diagnosed with NSP at seven months scored lower on all of the Bayley Scales of Infant and Toddler Development than the 231 children without NSP at seven months. The largest differences were seen in cognition, language, and parent-reported adaptive behavior. Even children with at least mild NSP at 36 months, which had not previously been detected—control group children—had lower developmental scores than unaffected children. However, these findings do not imply that NSP causes developmental problems [15]. In a follow-up study of 129 infants diagnosed in infancy with NSP, 11% had one or more delays in the parent-completed age-appropriate Ages and Stages Questionnaires at age 3–4, and 13% of parents reported concern [16]. A positive association between NSP and developmental delay was found in 13 of the 19 studies in a systematic review [17]. However, the severity of NSP cannot be used to predict the presence or degree of developmental delay according to findings of a prospective, nonrandomized study of 27 infants referred to a cranial facial clinic [18]. Thus, the relationship between NSP and early developmental delays remains poorly understood [19].

In a systematic review of 22 studies with a total population of 27,782 children, 60 risk factors for NSP were identified. The most commonly reported risk factors in these studies were: male, supine sleep position, limited neck rotation or preference in head position, firstborn, lower infant activity level, and lack of tummy time [20]. A prospective cohort study of 200 infants in the first two years of life found that three factors deter recovery: supine sleep position, limited head rotation, and lower infant activity level [6].

In 2008, a project was initiated in Skaraborg, Sweden in an attempt to prevent NSP. Sweden has a National Child Health Care Program, and the attendance rate was nearly 100% in 2005 [21]. The primary health care providers at the child health clinics are public health and/or pediatric nurse specialists. They are responsible for monitoring infants’ growth and development and informing parents about the Swedish Board of Health and Welfare’s recommendations, including recommendations on safe infant sleep positioning. Since nearly all infants in Sweden attend the child health clinics, these clinics provide an ideal venue for monitoring infant cranial shape and providing NSP prevention recommendations to parents.

The project commenced with a literature search on NSP prevention practices to develop evidence-based guidelines for the nurses [22]. The idea was to provide a working tool on NSP prevention for the busy nurses. The guidelines were tested in a pilot study [23] and revised. A continuing education on NSP, which included the revised guidelines, was developed for the nurses, and a clinical intervention was planned.

The overall aim of the dissertation [24] is to assess whether it is possible to prevent NSP while still promoting safe infant sleep practices. Specific aims include evaluating reliability of assessors judging infant cranial asymmetry in order to evaluate if they could be considered reliable and interchangeable assessors in the planned clinical intervention [25]; comparing intervention and control group parents’ responses regarding prevention recommendations they had received from their nurses during their infant’s early months [26]; assessing what knowledge from a continuing education intervention and control group nurses imparted to parents and parents implemented in their infant care [27]; evaluating the effect of the clinical intervention on infant head shape [5].

There are two research questions. Will educating child health nurses who, in turn, provide parents with tailored recommendations regarding infant positioning help prevent NSP? Can the intervention help reverse NSP that develops in early infancy?

## 2. Materials and Methods

### 2.1. Participants and Group Allocation

Five individuals unaffiliated with the child health clinics were recruited as assessors of infant cranial asymmetry for the clinical intervention. Of the child health nurses employed at the time, 79% agreed to participate. Fifty-three nurse specialists (35 intervention group and 18 control group), 274 newborns (182 intervention group and 92 control group) and their parents participated in the clinical intervention. The nurses, newborns, and parents were allocated to an intervention and control group. In an attempt to avoid a “spillover effect”, group allocation was based on whether nurses had previous exposure to the project from participating in the pilot study 2009 and/or attending the lecture on NSP held December 2010, or not. If no nurse at a child health clinic had previous exposure to the project, all nurses, newborns and their parents at that clinic were allocated to the control group; otherwise, the intervention group.

### 2.2. Setting

The reliability test–retest was mailed to homes of the five intended assessors prior to the intervention. The clinical intervention, survey, and qualitative inquiry were conducted at 26 child health care clinics in Skaraborg, Sweden.

### 2.3. Design

Strategies for NSP prevention were developed according to the five phases of evidence-based nursing practice: ask the clinical question, search for the evidence, critically appraise the evidence, apply the evidence to practice, and evaluate the effectiveness of the evidence [28]. A clinical intervention and three supporting studies—a reliability study, a cross-sectional survey and a qualitative inquiry—were conducted.

The reliability study was conducted prior to the clinical intervention. Assessors were taught how to judge infant cranial shape using two assessment tools—Severity Assessment for Plagiocephaly [29] and Severity Assessment for Brachycephaly [30]. Then assessors participated in a photograph test–retest in order to evaluate if they could be considered reliable and inter-changeable assessors for the clinical intervention. They judged infant cranial shape in 50 photographs of young children. They also participated in an infant test where they judged the cranial shapes of six infants.

Then a longitudinal clinical intervention with two arms was conducted. In the first arm, a continuing education on NSP prevention was provided for nurses at their own clinics prior to February 2012. The second arm, the clinical intervention itself, was launched in February 2012. Intervention group nurses had been educated about NSP while control group nurses had not. Intervention group nurses were asked to work according to the guidelines [24] while control group nurses were not given new directives. Nurses in both groups recruited newborns to the study by obtaining written informed parental consent.

**Figure 1:**
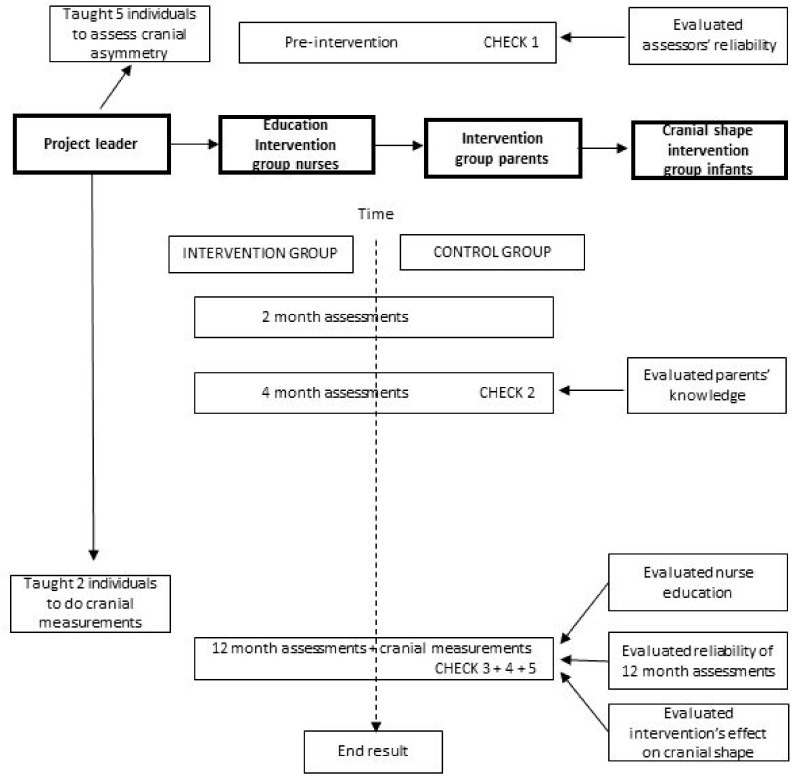
Design of the project.

Nurses in both groups continued to follow the National Child Health Care Program’s visits. Since capitalizing on the plasticity of the neonatal cranium is the essence of intervention [31], the intervention began for each individual infant and parent when their child health nurse came for the first home visit and received “This is Your Child’s Health Book” [32]. In order to evaluate how the clinical intervention had influenced cranial shape, infant cranial shape was assessed by the reliability-tested assessors in conjunction with each infant’s 2-, 4-, and 12-month child health visits, and cranial measurements were taken at their 12-month visit as well. The intervention continued until infants’ 12-month visits (Figure 1) [24].

A cross-sectional survey was conducted in conjunction with 4-month assessments in order to examine what knowledge parents received from their nurses during early infancy. In the survey, parents were given a checklist with 22 statements and asked to put a check in front of all the statements that pertained to information they had received from their nurse. Although some statements pertained to parents’ opinions, most statements pertained to aspects of NSP prevention considered beneficial for parents to be aware of during their child’s early infancy. This knowledge stemmed from “regular” recommendations (both national and child health program recommendations) and “new” recommendations (recommendations newly introduced in the continuing education for nurses). Some statements were worded positively and some negatively in an attempt to avoid response bias.

The continuing education for nurses was evaluated when infants were 12 months old. A qualitative inquiry was conducted to explore what knowledge had been integrated in practice. Intervention and control group nurses and parents were asked two open-ended questions regarding what they did to prevent and to reverse NSP.

### 2.4. Data Collection

In the reliability test–retest, 50 photographs of young children’s heads were sent by mail to the five assessors, assessed, and both photographs and results were returned by mail to the project leader. This procedure was repeated after at least one-week had elapsed and with the photographs in a different order to decrease recall. The five assessors also assessed the cranial shape of six 2-month old infants at one clinic on the same day. One assessor was in the room at a time with each infant and parent.

Cranial asymmetry of each infant was assessed in conjunction with their 2-, 4-, and 12-month visits by assessors blinded to group. Assessors were considered interchangeable and did not follow specific infants. In addition, four cranial measurements—cranial length, cranial width, and the two transcranial diagonals—were recorded at 12-month visits by individuals trained to use a craniometer (Infocefalia, Barcelona, Spain) and blinded to group. The individual taking cranial measurements was not in the room at the same time as the assessor.

Assessors asked parents to fill in a form on infant characteristics and care practices in conjunction with 2-month assessments. In conjunction with 4-month assessments, assessors asked parents to fill in a checklist regarding information they had received from their nurses. When infants were 12-months old, the project leader sent nurses a form by mail with two-open ended questions regarding what they did to prevent and reverse NSP. Nurses filled in the form and returned it to the project leader. In conjunction with 12-month assessments, assessors asked parents to fill in a form with two-open ended questions regarding what they did to prevent and reverse NSP. Data on infant safety were included in the checklist at four months and in the responses to open-ended questions at 12 months.

### 2.5. Analysis

Intra-assessor and inter-assessor reliability were analyzed using Agreement Coefficient 2 (AC2) with quadratic weights. Knowledge that parents in the intervention and control groups received from their nurses during early infancy was compared using Fisher’s Exact Test. Knowledge from the nurse education that had been integrated in practice was evaluated by comparing nurses’ and parents’ responses in the intervention and control groups in a qualitative content analysis and a case-by-case analysis with a process-oriented approach. A rating system was devised, and infant cranial shapes were compared in the intervention and control groups at 2, 4, and 12 months with the help of time series analysis, Chi^2^-test, risk ratios, AC2 with quadratic weights, logistic regression, and multiple logistic regression analyses (Table 1) [24].

## 3. Results

### 3.1. Reliability

Mean percentage of perfect intra-rater agreement was 73 in the photograph test-retest. Adjusted mean intra-rater AC2 was 0.69 [0.63; 0.76] and adjusted inter-rater AC2s were 0.72 [0.64; 0.81] and 0.71 [0.63; 0.79]. Adjusted inter-rater AC2 was 0.74 [0.60; 0.87] in the infant test. These results indicate the substantial strength of intra- and inter-rater agreement when interpreted according to Landis and Koch’s strength of agreement intervals [33]. Furthermore, the assessors’ detection of asymmetry in the infant test corresponded with the reference-rater in 23 of 24 instances, which is nearly perfect. Therefore, these five individuals were considered reliable and interchangeable assessors of cranial asymmetry assessments in the clinical intervention.

### 3.2. Follow through

Ninety-three percent of the participating nurses followed through. All infants and parents followed through unless the family moved—278 of 284 (98%) infants and their parents followed through, and six families moved.

### 3.3. Parents’ Knowledge of NSP Prevention During Infants’ Early Months

A significantly higher proportion of intervention group parents compared to control group parents were aware of three national recommendations at the time—alternate direction of the infant’s head when putting the child to bed (82%: 64%, *p* = 0.001), which pillow to use (92%: 80%, *p* = 0.01), and when to remove the pillow (48%: 31%, *p* = 0.006—and five of the seven new recommendations that were introduced to the nurses in the continuing education. The five recommendations newly introduced to nurses include the following: begin to introduce awake infant to tummy time by two weeks of age (51%: 34%, *p* = 0.007); limit time in an infant car seat to car rides (50%: 26%, *p* = ≤ 0.001); limit time in the infant bouncer (54%: 26%, *p* = ≤ 0.001); avoid subjecting the infant’s head to pressure from hard surfaces (67%: 42%, *p* = ≤ 0.001); and change arms when bottle feeding (54%: 17%, *p* = ≤ 0.001) (II). These results indicate that intervention group parents were significantly more aware of both national and newly introduced recommendations than control group parents during the early months when the infant’s cranium is most vulnerable to NSP development and has the greatest chance for NSP to reverse. In addition, the proportion of intervention group compared to control group parents that reported receiving verbal information from their nurse during their infant’s first four months was 96% vs. 85% (*p* = 0.002), and the proportion that reported receiving written information was 68% vs. 58%, although this observed 10% difference between groups is not statistically significant. Thus, verbal information was important to parents in the study. Five control group parents did not report having received either verbal or written information from their nurse, whereas all intervention group parent reported at least one of these.

### 3.4. Knowledge Integrated into Practice

Knowledge was integrated into practice by both nurses and parents. Intervention group nurses reported providing regular and new re-positioning recommendations to parents, including how to accomplish occipital pressure relief when infants are awake, asleep, and being fed. Intervention group parents reported implementing both regular and new recommendations in their infant care. Furthermore, intervention group parents who perceived severe cranial asymmetry at 3–4 months reported implementing regular and new re-positioning strategies in their reversal efforts.

### 3.5. Infant Safety Recommendations

Most parents in both groups reported that their nurse provided information on why an infant should have tummy time under surveillance (85%: 82%; *p* = 0.601) and most reported having been informed about which infant pillow is appropriate to use (92%: 84%; *p* = 0.001). However, less than half of the parents in both groups reported having been informed when to remove the pillow (48%: 31%; *p* = 0.006). Moreover, less than half of the parents in both groups reported having received an explanation regarding why they should remove the pillow (45%: 29%; *p* = 0.009). Furthermore, most nurses and parents that reported tummy time did not report the safety details of tummy time—that infants are awake and under surveillance while they are prone. Additionally, some parents in both groups reported placed infant prone for sleep, which is also a risk factor for SIDS. Moreover, the code *side sleep*, a risk factor for SIDS, is seen in both nurses’ and parents’ lists in the qualitative content analysis. These results indicate possible infant safety issues.

### 3.6. Infant Characteristics and Care Factors

The two infant groups were similar regarding birth-related risk factors. At two months, a larger proportion of intervention group infants compared to control group infants had parent-reported side preference (44%; 36%, *p* = 0.18); a smaller proportion of intervention group infants compared to control group infants were solely bottle-fed (21%; 35%, *p* = 0.01). There was a wide range in parent-reported time infants in both groups spent in positional devices daily, a care factor. In the intervention group, the minimum–maximum time infants spent daily in a bouncer was 0 min to 8 h, and in the control group the range was 0 min to 9 h 40 min.

### 3.7. The Effects of the Intervention on Infant Cranial Shape

In a longitudinal analysis, the proportion of intervention group infants with NSP was lower than control group infants at 2 months (T1), 4 months (T2), and 12 months (T3), but did not show a significantly different course of development. However, in a longitudinal analysis of the subgroup of infants with combined plagiocephaly/brachycephaly, intervention group infants showed a significantly different course of development than control group infants (*p* = 0.04), but the subgroup was small.

The prevention effect was estimated by considering the opposite of prevention—i.e., the occurrence of prevention failures—although what was actually prevented through our intervention instead of other sources is unknown. Non-cases at the outset of each time were the starting point. In the subgroups of infants who were non-cases at T1, 4% of intervention group and 11% of control group infants developed plagiocephaly, and 25% of the intervention group and 22% of the control group infants developed brachycephaly between T1 and T3. Nine intervention group and two control group infants developed brachycephaly after T2. Overall brachycephaly prevention failure (24%) was three times more common than overall plagiocephaly prevention failure (8%). It turned out that NSP was difficult to prevent in both groups, especially brachycephaly.

As for the reversal effect, there was a 24% difference between groups (65% intervention group; 41% control group) in NSP reduction from T1 to T2 (*p* = 0.06), i.e., considered early reversal, and a 50% difference between subgroups (50% intervention group; 0% control group) in combined plagiocephaly/brachycephaly reduction from T1 to T2 (*p* = 0.03). Yet plagiocephaly and brachycephaly reductions from T1 to T2 are not statistically significant. There was a 10% difference between groups in NSP reduction from T2 to T3. This is not statistically significant, indicating that the intervention contributed little to late reversal. Six (3%) intervention group and nine (10%) control group infants had NSP at each of the three assessments—i.e., persistent asymmetry, (RR = 0.35 [0.13; 0.94], *p* = 0.03)—indicating that belonging to the intervention group lowered infants’ risk for persistent NSP nearly threefold.

Factors that might help explain reversal were investigated. It was four times more common that NSP at two months reversed by four months in intervention group than in control group infants (OR = 4.07 [1.23; 13.44], *p* = 0.02) when adjusted for parent awareness of written recommendations from their nurse. However, it was nine times more common that NSP at two months reversed by four months when parents were aware of written recommendations from their nurse (OR = 9.09 [0.02; 0.48], *p* = 0.004) when adjusted for group. Additionally, it was >5 times more common that NSP at two months reversed by 12 months when parents were aware of written recommendations from their nurse (OR = 0.19 [0.04; 0.95], *p* = 0.04. Thus, it turned out that parents’ awareness of written NSP prevention recommendations from their nurse had more effect on reversal than the intervention itself.

Thirteen of the 15 infants who failed to reverse had ≥2 of the following risk factors: firstborn, side preference at T1, solely bottle-fed at T1, and spent ≥2 h daily in positional devices at T1. Birth-related factors, side preference, and care factors of these 15 infants were compared with those of infants whose NSP did reverse between T1 and T3. A larger proportion of infants whose NSP failed to reverse were firstborn (60% vs. 44%), had a vacuum-assisted delivery (27% vs. 11%), and were solely bottle-fed at T1 (53% vs. 33%). Moreover, their median daily bouncer time at T1 was higher (90 min vs. 30 min). However, these results are not significant. Thirteen of the 15 infants who failed to reverse had brachycephaly at T3.

## 4. Discussion

The assumption was that if child health nurses participated in a continuing education on NSP, were provided with guidelines to follow, and in turn provided tailored recommendations to parents of newborns, nearly all NSP would be prevented. Findings indicate that, while the intervention helped reverse NSP which developed in early infancy, it did not succeed in preventing NSP from developing. Examining what turned out to be successful or less successful in the project provides some useful insights for further prevention and reversal efforts.

Several strategies worked well. Motivation: Both nurses and parents were motivated to try to prevent NSP. Of the child health nurses employed at the time, 79% agreed to participate in the studies, and 93% of the participating nurses followed through. All parents followed through unless the family moved—278 of 284 parents (98%) followed through. The Swedish child health care setting turned out to be an ideal venue for motivating both nurses and parents to participate in NSP prevention efforts.

Imparted knowledge to parents: Findings of the 4-month survey indicate that, while both groups of nurses worked to inform parents about NSP prevention, educating child health nurses about NSP did increase parents’ awareness of recommendations. Intervention group parents reported significantly more recommendations from their nurse than control group nurses during the early months of infancy when parents’ knowledge can influence infants’ head shape. Findings of the 12-month qualitative inquiry indicate that intervention group nurses imparted both regular and new re-positioning strategies to parents, including how to accomplish occipital pressure relief when infants are awake, asleep, and being fed.

There are similarities between our 4-month survey and a qualitative nursing study from the UK [34]. That study was similarly conducted alongside an intervention study, and the nurses were also allocated to an intervention group that received an education or a control group which did not. Furthermore, these researchers also found that nurses in the intervention group actively applied their new knowledge and that nurses in the control group aimed for positive change by using their existing skills and experience.

Integrated new knowledge into practice: Intervention group parents’ responses to the open-ended questions regarding infant care included details of NSP prevention that were introduced in the continuing education for nurses; intervention group parents who perceived severe cranial asymmetry at 3–4 months reported utilizing newly introduced positioning strategies.

Another indication of integration of new knowledge into practice is the intervention group’s early reversal success of combination plagiocephaly/brachycephaly cases. This could be due to the continuing education for nurses including specific reversal recommendations while the national recommendations did not. Another reason could be that intervention group nurses learned how to assess cranial asymmetry, while control group nurses did not. A further indication is that intervention group nurses seemed to have integrated cranial asymmetry assessments in daily practice using the Severity Assessments because they provided assessments for 179 infants of the 184 intervention group infants at 2 months, and 180 at 4 months. However, we do not know if the nurses continue to make recommendations and do assessments now that the study is over.

Joint reversal efforts: Findings of the process-oriented approach in the qualitative inquiry indicate that nurses and parents collaborated in their attempts to reverse incipient NSP.

Decreased risk for persistent asymmetry: The risk for persistent asymmetry at 12 months was significantly lower for the intervention than the control group infants (RR = 0.35, [0.13; 0.94], *p* = 0.03) in the subgroup of infants who had NSP at two months. This indicates that intervention group nurses’ and parents’ collaboration was effective in decreasing infants’ risk of having persistent asymmetry at 12 months, although the numbers were low, i.e., six intervention group and nine control group infants.

Assessing cranial asymmetry: The findings indicate the substantial strength of assessor agreement when assessors were trained how to assess cranial asymmetry using Severity Assessments and then tested. Additionally, assessors showed excellent ability to detect NSP in the clinical setting. This indicates that their assessments in the clinical intervention can be considered reliable. In a wider clinical context, results indicate that child health nurses can also be trained to assess NSP, which can be helpful for early detection.

However, not all strategies were as successful. Intervention group nurses’ cranial asymmetry assessments did not always agree with assessors’, although both were trained to assess cranial shape in the same way. A sensitivity analysis of data intervention group nurses provided from their 2-month cranial asymmetry assessments showed a 65% sensitivity in detecting NSP when using assessors’ 2-month assessments as the gold standard. Intervention group nurses failed to detect 59% of cases detected by assessors. However, it is worth noting that intervention group nurses detected at least mild asymmetry in 31 of the 37 2-month assessor-detected cases, but mild asymmetry did not meet our rating system’s criteria for NSP. Moreover, comparing nurses’ assessments with those of the assessors is not completely fair because the assessors’ only duty was assessing cranial asymmetry, just one small part of the nurses’ job during a visit. Furthermore, the Severity Assessments are not precise tools.

Recommendations did not “get through” to all intervention group parents. Although all parents received the “This is Your Child’s Health Book” from their nurse, which included the regular recommendations, only 68% of intervention group parents reported having received written recommendations from their nurse during their infants’ first four months. Recommendations on infant positioning devices did not seem to get through to all intervention group parents either. Only 50% reported having received information on using infant car seats only during car rides, and only 54% reported having received information to limit time in infant bouncers. Parent-estimated minimum–maximum time spent in a bouncer daily was 0-480 min. Furthermore, only 48% of intervention group parents reported having received information from their nurse regarding when to remove the recommended infant pillow and why to remove the pillow, important safety aspects when providing an infant pillow. Some parents reported placing their infants prone or on the side for sleep, both considered unsafe sleeping positions. Furthermore, it is unclear how many intervention group parents were even aware of the need for surveillance during tummy time, because few reported this safety aspect.

In a qualitative study examining parents’ views of NSP prevention in Australia, researchers reported that some parents were more concerned about preventing NSP than SIDS because NSP was more real to them. Once NSP occurred, the majority of parents stopped following Australia’s SIDS guidelines on safe infant sleep [35]. In contrast, we did not observe parent incompliance to SIDS guidelines in our study. Few parents in our study reported unsafe infant sleep positions; parents who reported placing their infants prone for sleep provided explanations which indicate *unawareness* of SIDS guidelines, not incompliance [27].

Prevention was difficult in both groups, especially brachycephaly, despite nurses’ and parents’ high motivation to participate and follow through, and intervention group parents’ seemingly good knowledge about NSP prevention at four months. In the sensitivity analysis of the intervention group nurses’ 2-month assessments using assessors as the gold standard, nurses failing to detect about three in five cases at two months is one possible explanation for early prevention failure. In the subgroup of infants who were non-cases at T1 and subsequently developed brachycephaly, overall brachycephaly prevention failure in the intervention group (25%) was ≥6 times more common than overall plagiocephaly prevention failure (4%).

An Italian cohort study including 283 infants, reported that an estimated 38% of infants had plagiocephaly at two to three months and 12% had combination plagiocephaly/brachycephaly [36]. This is in contrast to our findings where the proportion of infants with plagiocephaly at two months in the intervention and control groups were 13% and 14%, respectively, and the proportion with combination plagiocephaly/brachycephaly were 5% and 7%. Although the results of a cohort study conducted at 2 to 3 months should not be compared with an intervention study conducted at 2 months, our control group results at least give an indication that the Swedish child health program provides a good starting point for NSP prevention.

In a randomized controlled trial (RCT) evaluating early intervention, recommendations were provided directly to parents by a neonatologist in a 15-min private guidance session and in written form before discharge from the maternity unit; NSP was assessed using 2D and 3D craniofacial imaging [37]. In this RCT, the prevalence of NSP was 11% in the intervention group and 31% in the control group in a 2D analysis at *three* months. In our study, the prevalence of NSP was 23% in the intervention group and 32% in the control group at *four* months using the Severity Assessments. When comparing net results, this age difference is important to consider, since NSP peaks at about four months [6]. In their follow-up study, where all parents concerned about their infant’s head shape received advice on repositioning regardless of previous group allocation, the head shapes of infants from three to 12 months were investigated. When sorted according to original group allocation, 13% of intervention group infants and 20% of control group infants in that study had NSP at 12 months [38], while 13% of intervention group and 16% of control group infants had NSP at 12 months in ours. Since the Severity Assessment is not nearly as accurate as 3D and 2D analyses, the results of these studies cannot be accurately compared. However, at least we seemed to do just as well when child health nurses provided parents with NSP prevention and reversal recommendations.

Early identification of head positional preference was missed in the continuing education and guidelines for nurses. Intervention group nurses were trained to evaluate the cervical range-of-motion in infants who were old enough to support their heads and were only instructed to ask parents about side preference. However, according to Rogers, 2011, the most important risk factor to find out about is whether an infant has a head positional preference. Rogers recommends asking parents about head positional preference at the first well-child visit and evaluating the cervical range-of-motion early—i.e., with neonates lying supine [39]. Asking parents specifically about head positional preference could help in the early identification of risk for developing brachycephaly, which turned out to be difficult in both prevention and reversal

## 5. Update

Providing an infant-adapted pillow for supine sleeping infants until they began to turn over was a recommendation in the clinical intervention, in accordance with recommendations at the time. In late 2013, after the study came to a close, the pillow recommendation was removed because there was no evidence that pillows helped prevent NSP (Appendix A). It is not known how pillows influenced cranial head shape in the clinical intervention.

## 6. Clinical Implications

The main principle of NSP prevention—to relieve pressure on the infant’s malleable occiput—is simple but important since newborns sleep a lot and lack muscle strength to change their own head position. Yet the supine sleep position which puts consistent pressure on an area of the infant’s occiput is recommended as the safest infant sleep position, and no infant should ever come to harm from NSP prevention and reversal efforts. Thus, infants’ vulnerability for NSP and the supine sleep position are both here to stay. Therefore, nurses need to intensify efforts to help parents understand the importance of reducing pressure on the occiput whenever infants are awake.

Interestingly, parent awareness of written recommendations from their nurse helped reverse NSP regardless of group, yet parents in the study seemed to remember receiving verbal information more than written information. Consequently, it seems as though both written and verbal information from their nurse are important for parents in NSP prevention efforts. Synthesizing these findings infers that nurses discussing recommendations when providing printed material could improve parents’ recall and understanding. However, recommendations need to be tailored to parents understanding and the situation at hand, so good communication skills are important.

Preventing NSP is a continuing challenge for several reasons: supine sleeping young infants will always be vulnerable to NSP; the flow of information from a nurse education to the actual integration of the many small recommendations into daily infant care is long, so information can get lost during the process; communication is complex.

## 7. Future Perspectives

Since parents of newborns have many other things on their minds, an intervention introducing NSP prevention to expectant parents would be worth investigating. Furthermore, due to the challenges of prevention, more research is needed. Additionally, incorporating new NSP research findings into programs is needed to move forward. For example, in a study of 98 children with plagiocephaly, published 2020, infants with a small anterior fontanelle were found to have less reversal potential with re-positional strategies compared to infants with a large anterior fontanelle [40]. Incorporating anterior fontanelle measurements into NSP prevention programs could alert caregivers to which infants might have less reversal potential, so that they follow these infants’ cranial form vigilantly and provide tailored recommendations to their parents.

## 8. Conclusions

Assessors were considered reliable; educating nurses on NSP increased parental awareness of recommendations and promoted integration of newly introduced re-positioning recommendations in practice; the intervention was associated with early NSP reversal and reduced infants’ risk that NSP at two months persisted at 12 months. However, prevention was difficult, especially brachycephaly prevention. More research on NSP prevention is needed.

## Figures and Tables

**Table 1:** Overview of methods and analyses.

Study	Role	Design	Participants	Data Collection and Time Point	Analysis
I	Supportive	Reliability test	Assessors	Photograph test-retest and	Agreement Coefficient 2 with
	study			infant test, prior to clinical	quadratic weights
				intervention	
II	Supportive	Cross-sectional	Parents	Parent checklist in conjunction	Fisher’s Exact Test
	study	survey		with 4-month well-child visits	
III	Supportive	Evaluation study	Nurses	Nurses and parents asked two	Qualitative content analysis and
	study		Parents	open-ended questions when	Qualitative case-by-case analysis
				infants were 12 months old	with process-oriented approach
IV	Main study	Longitudinal	Infants	Cranial asymmetry assessments	Time series analysis, Chi^2^-test,
		clinical intervention	Nurses	in conjunction with 2-, 4-, and	Agreement Coefficient 2 with
		with 2 arms	Parents	12-month well-child visits	quadratic weights, logistic
					regression and multiple logistic regression

## Appendix A

### Appendix A.1. Prevention of Acquired Infant Cranial Asymmetry

#### Appendix A.1.1. Guidelines for Child Health Nurses

The recommendation to place infants on their backs for sleep led to a decrease in Sudden Infant Death (SIDS), so this sleep position recommendation must always be followed for infant safety. However, the back-to-sleep position can easily lead to a flat area developing on the back of infants’ formable heads in the early months since infants cannot turn themselves. Re-positioning strategies initiated in early infancy help to prevent this flattening.

1. Begin informing parents at the first home visit. Show parents the information on page 14 of the Child’s Health Book and talk about it.
2. Assess back of infant’s head shape regularly for at least 6 months
  - Observe head from above and looking down-bird’s eye view
    Is there centered flattening? one-sided flattening? ear misalignment?
  - Observe and palpate back of head for flattening
    A bald spot on one side could be a sign of one-sided flattening.
  - Observe left and right profile view for flattening.
    Does the head peak?
  - Observe the infant’s face.
    Does the face seem wide despite average head circumference?
    Does one side of the forehead bulge?
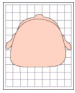
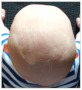
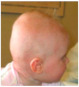
3. Assess for head positional preference and head tilt
  - Ask parent at first visit if their infant has a head positional preference.
    This can be counteracted by placing the infant’s head in another direction.
  - Ask parent if infant always looks in the same direction? This can be counteracted by encouraging the infant to look in the other direction.
  - Assess the cervical range-of-motion with the neonate laying down.
  - when infant can hold head upright, observe from the back for head tilt.
4. Monitor head shape carefully if infant has following risk factors: premature, firstborn, twin, side preference, torticollis, only bottle-fed, vacuum-assisted delivery, born with a flat spot, cephalohematoma, decreased movement or level of activity, and brace for hip dysplasia.
5. Refer to physician: if asymmetry does not improve within 2 months; if craniosynostosis is suspected; if torticollis is suspected.

#### Appendix A.1.2. Recommendations for Parents to Prevent Cranial Asymmetry

Infants should sleep on their back and lie with their head alternatingly in the right and left direction.

Minimize pressure on the back of infant’s head whenever infant is awake.

- Begin getting infant accustomed to tummy time by first week.
- For safety, the infant should always be awake and under surveillance during tummy time.
- Begin with short periods of tummy time several times daily and increase the time successively.
- Make tummy time fun by being on the floor together or placing a toy in front of your infant.
- Pick up your infant before the child becomes too upset.
- An infant can also become accustomed to tummy time by laying on the chest of a reclining parent or by laying on a parent’s lap.
- If you place your infant’s elbows close to their body, this will provide a more stable base when your infant begins to lift his/her head.
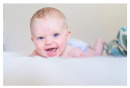
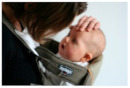
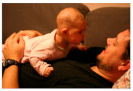

- Only have infant in car seat during car rides.
- Minimize time infant spends in bouncer and swing.
- Minimize time back of infant’s head is placed on hard surfaces, such as on the floor in a stationary activity center.
- Alternate arms when bottle-feeding.
- Carry your infant.

#### Appendix A.1.3. Recommendations for Parents to Reverse Flattening that Has Developed

The major principle is to alleviate pressure on the back of infant’s head whenever infant is awake.

- Increase tummy time under surveillance as much as possible when infant is awake.
- Carry your infant in your arms or in a carry shawl.
- The Bumbo chair is useful once your infant has begun to sit, but your infant needs to be watched and should only sit as long as he/she can hold his/her head upright.
- Avoid longer periods in car seats, bouncers, and infant swings where your infant’s head is not supported, otherwise a side preference can develop quickly.
- For infants who are bottle-fed and have developed a one-sided flattening, change arms so that the flattened side is upwards.

To alleviate pressure on the flattened area while the infant sleeps, use Delta Baby side pillow (wedge pillow) in the crib and carriage. This wedge pillow provides safe side positioning. If it is one-sided flattening, the flattened side of the head should be upward when placed for sleep. If the flattening is in the middle, alternate left and right side upward when placed for sleep.

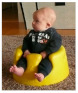
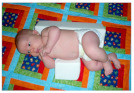

Developed by Freda Lennartsson, Skaraborg, Sweden 2018 in consultation with Professor Göran Wennergren.
